# Digital Medicine in the Management of Heart Failure: From Reactive Care to Predictive, Pathophysiology-Driven Strategies

**DOI:** 10.3390/healthcare14040455

**Published:** 2026-02-11

**Authors:** Ulvi Mirzoyev, Kanan Mirzoyev

**Affiliations:** 1 Melhem International Hospital, 1099 Baku, Azerbaijan; 2 Department of Cardiology, Libera Università Vita-Salute San Raffaele, 20132 Milan, Italy

**Keywords:** heart failure, digital medicine, remote patient monitoring, telemedicine, arrhythmias, artificial intelligence, pulmonary artery pressure, patient-centered care

## Abstract

Background: Heart failure (HF) is a progressive, multisystem syndrome characterized by recurrent decompensation, high hospitalization rates, and substantial mortality. Conventional HF management is mainly episodic and often fails to detect worsening conditions in advanced disease. Digital medicine and remote patient monitoring (RPM) hold promise for advancing HF care by enabling earlier detection, proactive action, and personalized care. Methods: We conduct a narrative review to summarize evidence from randomized clinical trials, real-world registries, and emerging digital health technologies regarding the present and future utility of digital medicine in HF care. There is greater emphasis on pathophysiology-based surveillance, personalized care models, and integration into planned health care pathways. Results: Integrated digital interventions, such as implantable hemodynamic monitoring, organized telemedicine programs, or device-based diagnostic technologies, can minimize HF hospitalizations, prolong life, improve quality of life, and optimize resource utilization in health care systems when incorporated into coordinated care. Crucially, trials emphasize that clinical benefit depends not on technology but on a prompt clinical response, multidisciplinary cooperation, and ongoing interaction between the patient and the doctor. New technologies—including voice-based biomarkers, smartphone-derived photoplethysmography, ballistocardiography, and artificial intelligence–driven data integration—may help transition RPM from a hardware-based system to a scalable, “deviceless” approach. Conclusions: Digital medicine is a game-changer for reimagining HF care, involving not only continuous monitoring of physiological changes but also personalized, proactive clinical decision-making. To implement truly patient-centered, predictive HF management in the years to come, technological innovation must be combined with human connection, ethical governance, and health-system readiness.

## 1. Introduction

**Figure 1:**
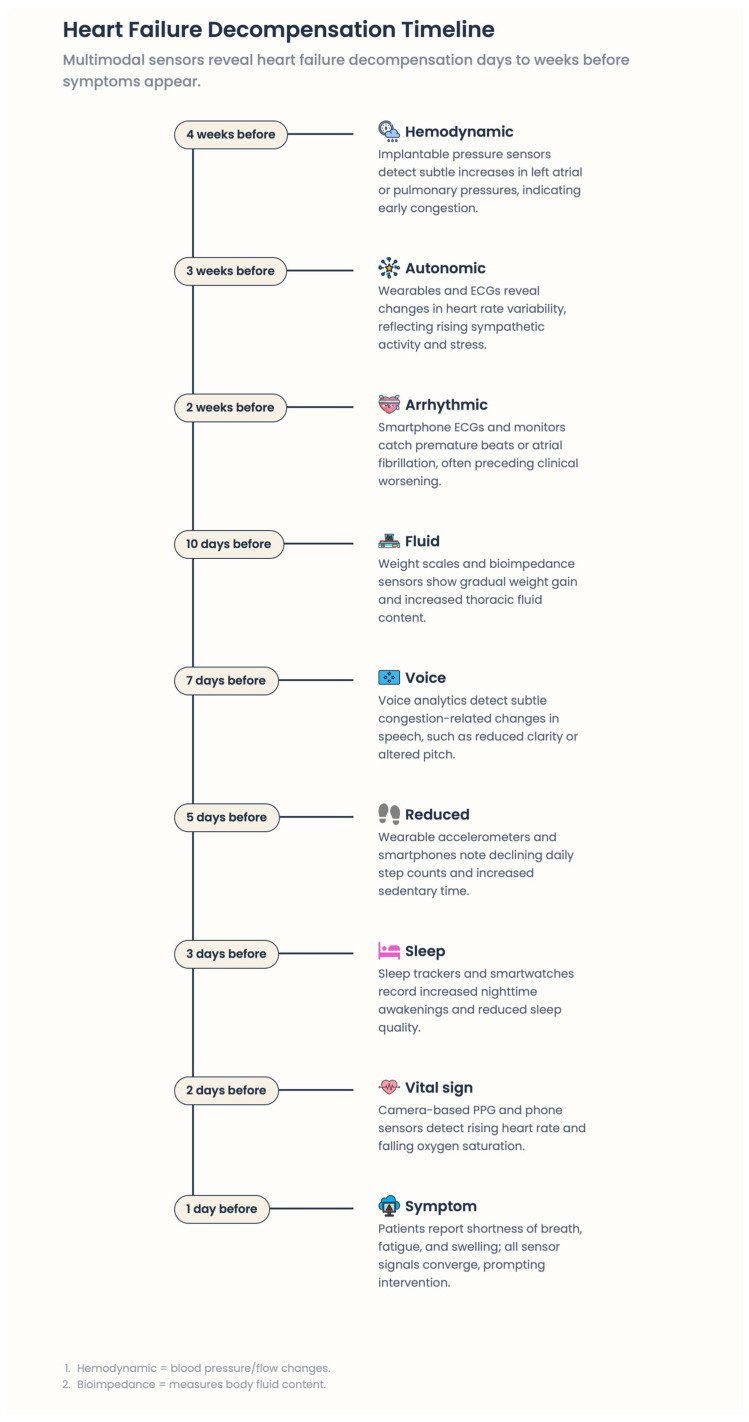
Heart failure decompensation timeline.

Heart failure (HF) is one of the world’s most complex and economically challenging cardiovascular syndromes and a common final pathway for numerous structural, functional, and electrophysiological heart disorders [1]. Despite significant progress in pharmacologic and device therapies, HF remains associated with substantial morbidity, frequent hospitalizations, poor quality of life, and high healthcare delivery costs. HF progresses over days or weeks (fluctuating hemodynamic status, neurohormonal activation, and altered arrhythmia risk) before an overt worsening of clinical status [2]. Nevertheless, traditional HF care models are primarily episodic and reactive, based on periodic clinical checks that often identify decompensation only after substantial pathophysiological deterioration has already occurred. The mainstay of HF care focuses on outpatient visits and self-reported presenting symptoms, with hospital-based interventions reserved for acute decompensation. This design also limits clinicians’ ability to detect, early in the patient’s course, subclinical changes in volume status, autonomic balance, or cardiac rhythm that can lead to worsening HF or malignant arrhythmias [1]. Therefore, many patients present late in the course of illness, when treatment is not readily available, hospitalization is a prerequisite, and prognosis is poor. In addition, breakdowns in care among primary care providers, cardiologists, and hospitals, along with suboptimal patient engagement and medication adherence, increase the likelihood of adverse outcomes [3,4]. Concurrently, HF pathophysiology has increasingly been recognized as an ongoing, measurable process rather than a sequence of discrete clinical episodes. Hemodynamic congestion, progressive ventricular remodeling, neurohormonal dysregulation, and autonomic disturbance occur slowly and may be characterized by physiological signals long before clinical symptoms become evident [5] [Figure 1].

### 1.1. Review Design and Evidence Selection

This article was conducted as a narrative review, an approach appropriate for complex and rapidly evolving fields where interventions, technologies, endpoints, and care models are heterogeneous and not readily amenable to formal meta-analysis. The literature was identified through targeted searches of PubMed, Embase, and guideline repositories, focusing on digital health, telemonitoring, remote patient management, implantable and non-invasive monitoring, and heart failure outcomes. Evidence was selected for clinical relevance, mechanistic linkage to heart failure pathophysiology, and contribution to understanding implementation within structured care models. Digital modalities were categorized into invasive/device-based, non-invasive, and deviceless approaches, and evidence was interpreted in relation to clinical endpoints (hospitalizations, mortality, quality of life), workflow integration, and health-system impact. Emphasis was placed on landmark randomized trials, real-world implementations, and contemporary guideline perspectives.

### 1.2. Gap in the Literature

Although there is an emerging literature on digital technologies and remote monitoring tools for heart failure, most reviews are technology-centric, focusing only on what they see and do, and fail to integrate heart failure pathophysiology, clinical workflows, and implementation context into clinical considerations. Especially, a handful of reviews critically explore why some forms of digital monitoring have shown clinical utility in some respects while others have yielded neutral results, or how these physiological signals are translated into timely therapeutic action within real-world care pathways. This review aims to fill that gap by presenting a pathophysiology-informed synthesis of digital monitoring modalities in heart failure with a focus on connected-care models, implementation determinants, and value-based outcomes rather than technology alone.

Importantly, several of these processes are intricately associated with the pathogenesis of atrial and ventricular arrhythmias, which in turn influence the disease course, sudden cardiac death, and repeated hospitalizations [6]. This is one of the reasons to consider continuous monitoring strategies effective for detecting early changes from physiological stability. Digital medicine and remote patient monitoring (RPM) have emerged as promising tools for addressing these unmet needs, enabling longitudinal, real-time patient assessment outside the laboratory [7,8]. Sensor technology, implantable cardiac devices, telecommunication platforms, and artificial intelligence (AI) technologies have enabled continuous measurement of physiological parameters, including heart rhythm, heart rate variability, thoracic impedance, pulmonary artery pressure, body weight, blood pressure, and patient-reported symptoms. When embedded within structured care trajectories, these technologies may help transition HF care from a reactive, crisis-focused model to one that prioritizes early intervention and predicts clinical deterioration [9]. In addition to their technological aspects, digital health solutions would create a conceptual shift regarding HF care. Moreover, by continually monitoring the biological drivers of disease progression, digital medicine enables correlation of dynamic pathophysiological alterations with timely therapeutic interventions. This strategy aligns with modern preferences for personalized and precision medicine, in which treatment intensity, medication titration, and clinical follow-up are individualized based on risk profiles and changes in disease status [10]. Digital applications, when applied correctly, could also improve patient engagement, self-management, and adherence to guideline-directed medical therapy, thereby effectively addressing multiple non-biological determinants of HF outcomes. Digital medicine for HF is widely studied and adopted, but its clinical impact varies across testing and clinical practice. Variability across patient selection, monitoring strategy, clinical workflow, and healthcare system integration has yielded mixed findings, advocating for a nuanced interpretation of the available evidence [7,8,9,11]. Importantly, much of the literature has focused on clinical outcomes and has missed the opportunity to fully investigate how digital monitoring interfaces with the complex pathophysiology of HF and arrhythmias. More insights into these biological interactions are needed to ensure the optimal design, implementation, and follow-up of digital health efforts. We focus here on a narrative review of the current body of knowledge on digital medicine and remote patient monitoring for the treatment of heart failure, with particular attention to the pathophysiological mechanisms and implications of arrhythmias. We review the biological rationale for continuous monitoring, summarize key clinical evidence, describe possible integration into healthcare systems, and highlight current challenges and limitations. To wrap up, we discuss future perspectives toward predictive, personalized, pathophysiology-focused HF care driven by digital technologies.

## 2. Methods

### Characterization and Categorization of Digital Monitoring Parameters

The selected digital monitoring parameters discussed in this narrative review were chosen based on their relevance to the pathophysiological progression of heart failure and their feasibility for longitudinal remote monitoring. Instead of being strictly technology-driven, variables were organized into major areas of HF decline and associated clinical actionability. These domains comprised (i) congestion and hemodynamics (e.g., body weight, thoracic impedance, pulmonary artery pressure monitoring), (ii) markers of autonomic and cardiovascular reserve (e.g., heart rate, heart rate variability, trends in blood pressure, physical activity, and sleep patterns), (iii) electrical instability (e.g., detection of atrial fibrillation, ventricular arrhythmia burden), and (iv) symptom and functional status reported by the patient. The pathophysiology-driven framework was adopted to design the evidence synthesis and evaluate the translation of biological signals into actionable clinical responses via various digital modalities within structured care pathways.

## 3. The Pathophysiological Basis of Heart Failure Relevant for Digital Monitoring

**Figure 2:**
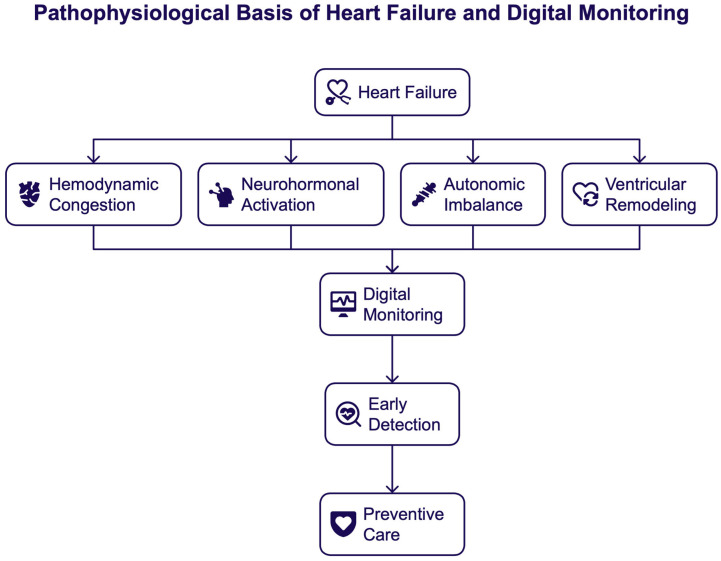
Pathophysiological basis of heart failure and digital monitoring.

Heart failure (HF) is a dynamic, progressive syndrome defined by complex, interlinked pathophysiological processes that are not static. More specifically, these processes include hemodynamic congestion, neurohormonal activation, autonomic imbalance, myocardial remodeling, and heightened predisposition to malignant arrhythmias. Crucially, most of these processes develop stealthily over days or weeks before patients exhibit signs of clinical deterioration. The temporal dissociation between pathophysiological deterioration and clinical manifestation is an essential weakness of long-standing, episodic models of HF care. It forms the biological underpinning for the need for ongoing, remote monitoring [12,13,14,15] [Figure 2].

### 3.1. Hemodynamic Congestion and Fluid Overload

Congestion is the primary pathophysiological feature of HF decompensation and the most common reason for hospitalization. High intracardiac filling pressures often occur without symptoms such as dyspnea, peripheral edema, or weight gain for a few days [16,17]. Subclinical elevations in left ventricular end-diastolic pressure and pulmonary capillary wedge pressure result in pulmonary and systemic venous congestion, impaired gas exchange, and progressive exercise intolerance. Standard outpatient treatment focuses primarily on patient-reported symptoms and transient physical exams, which are insensitive to early congestion. Consequently, treatment escalation often occurs late in the decompensation cascade. Digital technology for monitoring volume status surrogates (e.g., body weight trends, thoracic impedance, pulmonary artery pressure, and activity levels) enables detection of hemodynamic worsening before disease onset [12,13,14,15,16,17]. Pathophysiologically, early recognition of elevated ascending filling pressures allows early adaptation of diuretic therapy and neurohormonal blockade, which may delay progression to a clinical syndrome of full decompensation.

### 3.2. Neurohormonal Activation and Disease Progression

Long-term activation of neurohormonal systems, especially the renin–angiotensin–aldosterone system (RAAS) and the sympathetic nervous system (SNS), is central to disease progression in HF. Initially compensatory, persistent neurohormonal activation causes vasoconstriction, sodium and water retention, myocardial fibrosis, and adverse ventricular remodeling [18]. These processes not only worsen pump function but also contribute to electrical instability and arrhythmogenesis, increasing their likelihood. Activation of the neurohormonal system is closely linked to physiological parameters that can be indirectly measured using digital health technologies. Resting heart rate, heart rate variability, blood pressure trends, sleep quality, and physical activity patterns all reflect autonomic balance and cardiovascular reserve [19]. Persistent tachycardia, decreased variability, and reduced activity indicate increased sympathetic drive and disease instability. Frequent assessment of these conditions enables clinicians to better identify underlying biological stressors and optimize guideline-directed medical therapy (GDMT) with greater precision—particularly during titration.

### 3.3. Autonomic Imbalance and Arrhythmogenic Substrate

HF is characterized by marked dysregulation of the autonomic nervous system, as evidenced by elevated sympathetic tone and reduced parasympathetic activity. This imbalance underpins disease progression, compromises functional capacity, and predisposes to atrial and ventricular arrhythmias. Abrupt shifts in autonomic tone may precipitate atrial fibrillation, ventricular tachyarrhythmias, or electrical storms, often without warning [20,21]. Digital monitoring systems, particularly those with rhythm monitoring, wearables, or implantable devices, enable long-term monitoring of the heart’s electrical activity. Identification of increasing atrial ectopy, new-onset atrial fibrillation, or changes in ventricular arrhythmia burden could indicate worsening myocardial stress or congestion. From a pathophysiological perspective, arrhythmias are not simply anomalous events—they are harbingers of systemic failure, linking electrical abnormalities with hemodynamic and neurohormonal decline. Sequential rhythm monitoring thus serves as a bridge between the biology of arrhythmias and clinically actionable information [21].

### 3.4. Ventricular Remodeling and Progressive Myocardial Dysfunction

Structural modifications, including chamber dilation, wall thinning, fibrosis, and altered geometry, contribute to the gradual development of cardiac dysfunction in HF [22]. These changes develop over months to years and are mediated by recurrent episodes of congestion, ischemia, inflammation, and neurohormonal hyperactivity. Decreased exercise tolerance, reduced cardiac output, and increased arrhythmic risk are strongly related to cardiac remodeling. Although imaging modalities such as echocardiography and cardiac magnetic resonance remain the gold standard for structural evaluation [23], functional effects of remodeling can be continuously monitored using digital tools. Decreases in daily activity, walking distance, and the exertional heart rate response may indicate poorer cardiac reserve well before changes become detectable on scheduled imaging. Therefore, functional capacity phenotyping in a digital context supplements structural evaluation and captures the residual effect of myocardial remodeling on patients’ daily lives [24].

### 3.5. The Pre-Symptomatic Phase of Decompensation: An Opportunity

An essential concept in digital medicine for HF is the importance of an undetectable early stage of decline. Pathophysiological disturbances—increasing filling pressures, autonomic imbalance, greater congestion, and enhanced arrhythmic vulnerability—are already present and may persist during this period, yet patients may remain relatively well [25]. Conventional models largely overlook this stage because clinical contact is infrequent and reactive. Remote patient monitoring fills this void by translating continuous physiological signals into early warnings of impending instability. When real-time data streams are integrated with established biological pathways of HF progression, digital medicine facilitates a shift from crisis intervention to anticipatory, preventive care. This approach is grounded in pathophysiology, addressing the earliest manifestations of disease rather than later clinical effects.

### 3.6. Pathophysiology Underpins Digital Heart Failure Care

Recognizing HF as a biologically dynamic syndrome informs the framework for digital health interventions. In doing so, continuous monitoring not only generates data but also captures the temporal evolution of disease mechanisms that were previously available only between clinic visits. Remote monitors translate complex biology into actionable insights by linking digital signals to congestion, neurohormonal activation, autonomic dysfunction, and arrhythmogenesis [26]. Considering this, digital medicine must be considered not as an adjunct to HF treatment but as a complement to pathophysiological assessment outside the hospital and clinic. In the following sections, we will analyze how these biological concepts have been applied in real-world digital environments, the evidence that underpins their clinical value, and the future of digitally facilitated heart failure care.

## 4. Heart Failure: Digital Medicine Modalities

### 4.1. Structured Synthesis of Evidence by Technology and Endpoints

The evidence supporting digital health and remote patient monitoring in heart failure is summarized by major technology categories and associated clinical endpoints to enhance clarity and clinical relevance. These categories include (i) structured, non-invasive remote patient management programs, (ii) implantable hemodynamic monitoring systems, (iii) diagnostic data from implantable cardiac electronic devices, and (iv) wearable or deviceless digital monitoring technologies. Across these modalities, outcomes are interpreted with respect to mortality, heart failure hospitalization, composite endpoints, quality of life, and healthcare utilization, with consideration of patient risk profile and implementation context (Table 1).

Across modalities, clinical benefit is most consistently observed when digital monitoring is embedded in structured care pathways with predefined escalation and sustained patient–healthcare professional interaction. Technology alone is insufficient to improve outcomes.

**Figure 3:**
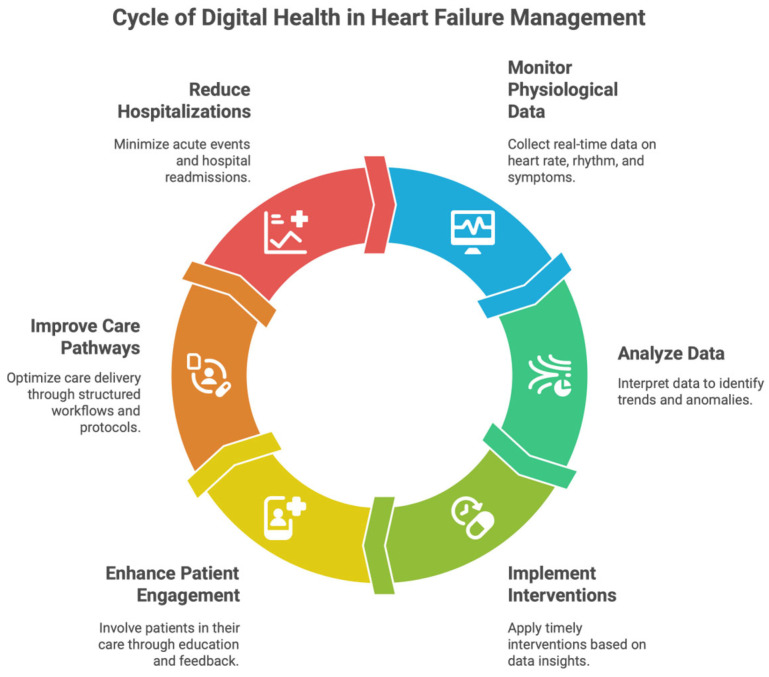
Cycle of digital health in heart failure management.

**Figure 4:**
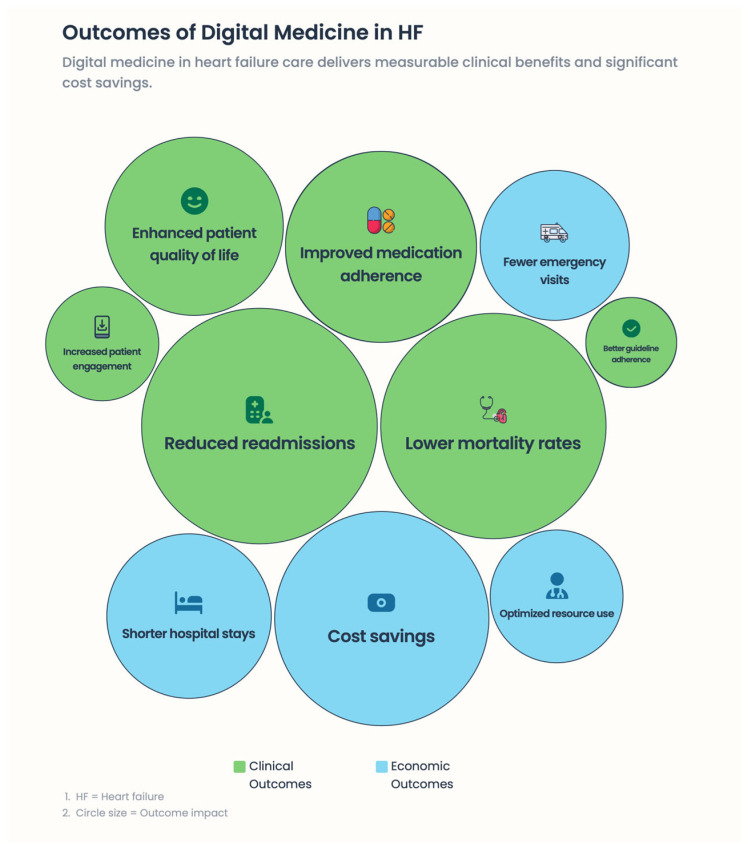
Outcome of digital medicine in HF.

Digital medicine in heart failure (HF) involves dynamic, advanced technologies that enable ongoing, rapid monitoring of medical status, early recognition of clinical deterioration, and delivery of targeted therapeutic interventions at optimal time frames [27]. Because traditional episodic care models have not extended to digital modalities—unlike episodic, one-off clinical encounters—digital modalities address dynamic changes in cardiovascular physiology, patient behavior, and treatment adherence in real-life settings. These tools also help move from reactive, event-based care toward predictability, an individualized approach to managing the disease, and a proactive approach [27] [Figure 3 and Figure 4].

### 4.2. Remote Patient Monitoring Systems

Remote patient monitoring (RPM) systems form the foundation of modern digital HF care. These systems are designed to integrate the routine transmission of physiological information (body weight, blood pressure, heart rate, rhythm, and symptoms) into a unified dashboard shared with health care teams. The primary objective of RPM is to identify subclinical deterioration in patients, specifically congestion and autonomic disturbance, before frank decline [28]. On a regular basis, small daily changes in weight, blood pressure, or symptoms generally lead to hospitalizations lasting days to weeks for patients with chronic HF. By using RPM to compare individual measures and longitudinal trends in these variables, clinicians recognize these trends early.

**Figure 5:**
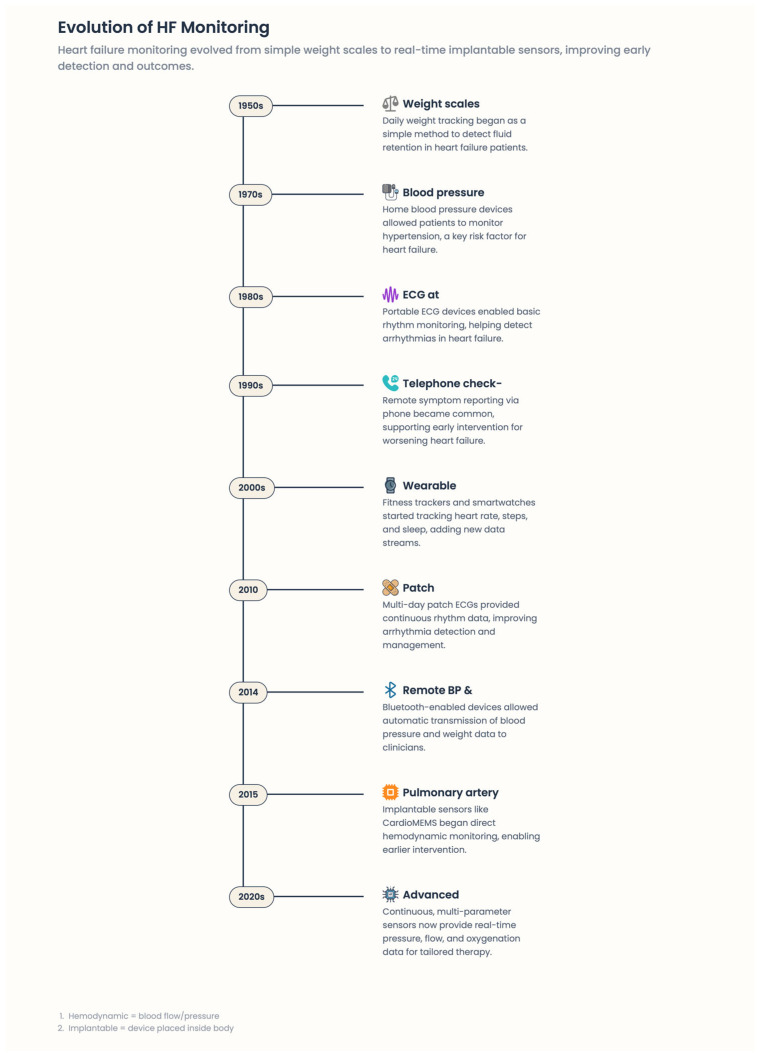
Evaluation of HF monitoring.

Furthermore, RPM applications support bidirectional communication, enabling patients to tailor therapy in real time, educate themselves, and re-cultivate self-management [29]. This communication element is what separates contemporary RPM from legacy telemonitoring approaches that focused on passively recording information [Figure 5].

### 4.3. Monitoring Based on Implantable Devices

Monitoring with implantable cardiac electronic devices such as ICDs and CRT systems provides continuous intracardiac data from implantable cardioverter-defibrillators (ICDs) and cardiac resynchronization therapy (CRT) systems [30]. In addition to their therapeutic role, these devices can track rhythm, heart rate variability, atrial arrhythmia burden, patient activity, and active exercise levels (across the heart-to-heart, chest, wrist, and thoracic distances), as well as atrial pulse parameters, chest wall, and respiration through device-derived respiratory parameters. Mean changes in thoracic impedance, for instance, indicate pulmonary fluid accumulation and are associated with future HF decompensation [31]. Likewise, a greater burden of atrial arrhythmia may indicate worsening atrial pressure, autonomic imbalance, or progression of structural heart disease [32]. Device-derived data can be incorporated into remote monitoring systems to detect early hemodynamic deterioration and increased arrhythmic risk, enabling clinical response. However, device-level alerts, when interpreted clinically in isolation, can lead to alert fatigue [33]. Thus, the most meaningful clinical information is delivered when device data are integrated into treatment pathways and combined with clinical, biochemical, and patient-reported information.

### 4.4. Hemodynamic Monitoring and Pulmonary Artery Pressure Sensors

**Figure 6:**
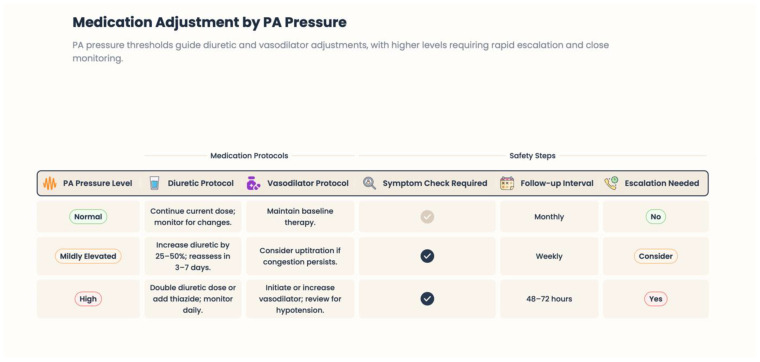
Medication adjustment by PA pressure.

Patient-directed hemodynamic monitoring is an essential advance in digital HF treatment. Implantable pulmonary artery pressure sensors provide continuous or periodic monitoring of intracardiac filling pressures, which can be detected early as HF wanes—if not before overt symptoms or weight gain become evident [34]. High filling pressures are a key pathophysiological driver of congestion, dyspnea, hospitalization, and mortality in HF [35]. Pulmonary artery pressure–guided management allows clinicians to proactively titrate diuretic and vasodilator therapy, thereby preventing acute decompensation [Figure 6]. This approach directly targets the hemodynamic substrate that promotes HF progression and has been successful in reducing hospitalization rates in targeted patient populations [36,37].

### 4.5. Wearable Technologies and Artificial Intelligence (AI) in the Management of HEART Failure Patients

The use of wearable and home-based non-invasive sensor systems is broadening the scope of digital HF care beyond implanted devices, offering more options and applications. These include smart scales, blood pressure monitors, wearable electrocardiography, photoplethysmography-based heart rate sensors, activity trackers, and sleep devices. These tools provide longitudinal information on functional capacity, autonomic control, circadian rhythm, and lifestyle [38]. Rising levels of physical activity and sleep performance are now recognized as essential determinants of HF stability and prognosis. Both low levels of activity and sleep disturbances indicate increased congestion, reduced cardiac output, or neurohormonal activation. Wearable technologies enable these gradual changes to be tracked in real time and linked to clinical outcomes, improving risk stratification and personalized care [39]. The main limitations of these approaches are the high heterogeneity in data accuracy, patient adherence, and platform integration. To achieve the maximum clinical impact, these technologies should be standardized and embedded into clinical practice—digital interventions for medication adherence and self-management [40]. High rates of medication non-adherence in HF patients are a leading cause of poor outcomes and are often overlooked in routine care. Digital medicine offers many solutions to this problem, including medication reminders, digital pillboxes, symptom checklists, educational applications, and more. These tools can enhance adherence to guideline-directed medical therapy while enabling patients to take an active role in their care [41]. Educational modules, personalized feedback, and reminders to call the health provider when thresholds are reached are often integrated into self-management platforms. Digital self-management tools address behavioral and cognitive aspects of HF care that are often poorly integrated or addressed in brief clinical encounters, reinforcing knowledge and promoting patient engagement [42]. This is done using artificial intelligence and predictive analytics. Advanced digital HF platforms increasingly rely on machine learning algorithms and artificial intelligence (AI). AI-driven systems seek to identify complex patterns in data—by aggregating numerous heterogeneous sources, including physiological signals, device diagnostics, clinical history, and patient-reported outcomes—that can predict decompensation, arrhythmias, or adverse consequences. Instead of using a single-parameter threshold, predictive models can generate risk profiles tailored to individual risk factors and early warning signals, thereby informing clinical planning [43]. Critically, however, systems such as these can shift the paradigm from merely monitoring threshold performance to dynamically predicting risk, thereby likely significantly changing HF management by better integrating the biological complexity of HF progression. However, transparency, interpretability, and clinical validation of AI models remain crucial to broad adoption [44]. Algorithms should support, rather than replace, clinical judgment and be embedded within care pathways that outline guidelines.

### 4.6. Integration of Digital Modalities into Comprehensive Care Models

All digital modalities differ in their benefits, but in practice, their primary value lies in their integration into an extensive system alongside an integrated HF care model. Effective digital HF management requires seamless communication among patients, primary care providers, specialists, and hospitals. Digital tools do not break down healthcare; they support it by enabling decision-makers to share responsibilities, deliver timely interventions, and ensure continuity of care across hospitals. In this context, digital medicine is no longer merely an auxiliary technology; it also enables system-level transformation [45]. When tailored to align with multidisciplinary HF programs, reimbursement pathways, and patient-centered care paradigms, digital modalities significantly affect disease course, reduce hospital admissions, and improve patients’ quality of life [46].

## 5. Evidence Base: Clinical Trials and Real-World Data—Why Some Digital Strategies Work, and Others Fail

### 5.1. Interpretation of Evidence and Sources of Heterogeneity

Support for digital health and remote patient monitoring in heart failure is heterogeneous and highly context dependent. Randomized trials have demonstrated that digital monitoring does not uniformly improve outcomes when deployed as passive data collection or without clearly defined clinical response pathways. In contrast, trials integrating structured care models with predefined escalation algorithms and continuous patient–healthcare professional interaction—such as TIM-HF2 and implantable hemodynamic monitoring studies—have shown reductions in unplanned hospitalizations and, in selected populations, signals toward improved survival. Importantly, these benefits cannot be extrapolated to all digital monitoring approaches or patient groups. Differences in patient selection, monitoring intensity, intervention thresholds, and health-system readiness represent key moderators of clinical effect. Real-world programs support these findings but also highlight variability in outcomes depending on workflow integration, staffing models, and patient engagement. Emerging modalities, including deviceless and AI-assisted monitoring, remain promising but require prospective validation before their impact on hard clinical endpoints can be established.

**Figure 7:**
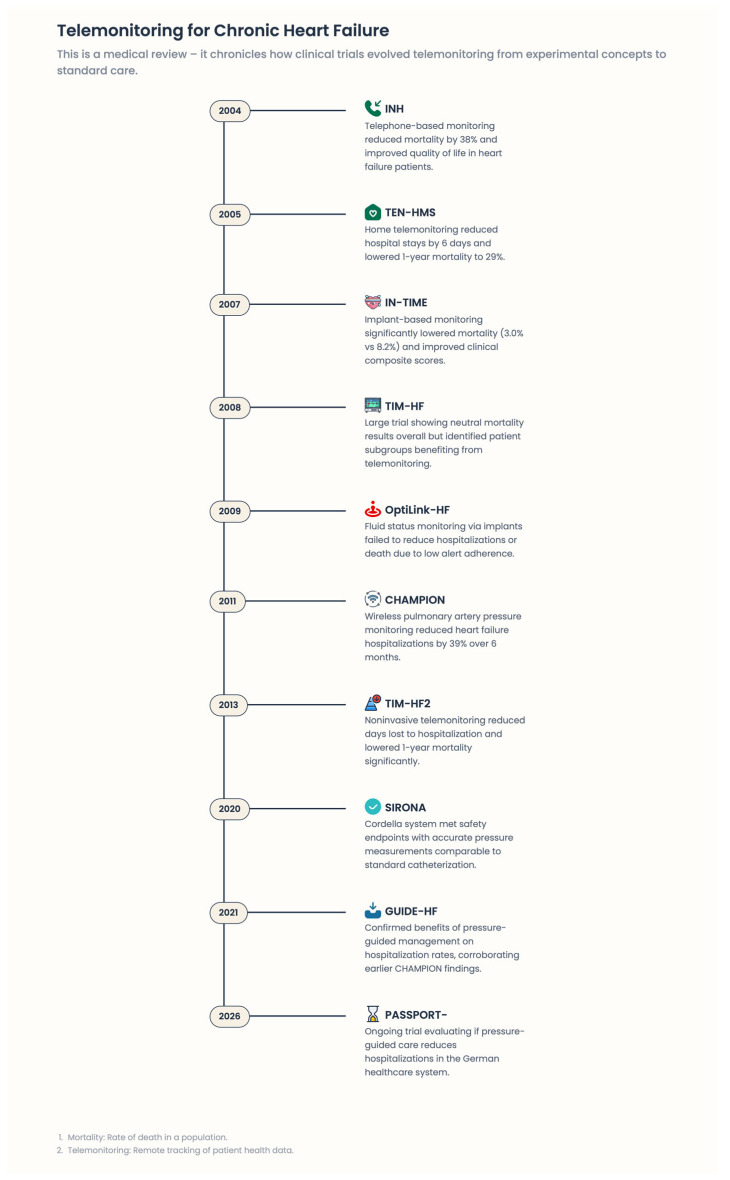
Telemonitoring for chronic heart failure.

Both digital medicine and remote patient monitoring (RPM) have been robustly studied in heart failure (HF) over the past 20 years, including randomized controlled trials (RCTs), pragmatic trials, and large-scale real-world databases [Figure 7]. These studies found similar advantages, though the efficacy of remote monitoring and digital therapies remained contentious. Although initial studies produced mixed, if not neutral, results, there is now a strong evidence base showing that digital interventions can work when based on pathophysiological principles, integrated into structured care pathways, and focused on ongoing patient–healthcare professional interaction. The disparate findings of these studies should be interpreted not as contradictions but as reflections of fundamentally different models of care.

### 5.2. Telemonitoring Trials in Their Early Days: Why Passive Digitalization Had Not Worked

Early telemonitoring trials primarily focused on remote detection of baseline clinical variables, such as weight and blood pressure, as well as patient-reported symptoms. These studies, including Tele-HF [47] and BEAT-HF [48], did not show significant reductions in mortality or HF hospitalizations. They created skepticism about the clinical utility of digital HF treatment, which remains neutral. However, a critical analysis shows that these early interventions had several limitations. Data transmission was usually passive, reviewed at intervals, and not linked to predefined therapeutic interventions [49]. Clinical workflows were rarely adjusted, there were no explicit escalation pathways, and accountability for responding to incoming information was not well defined. Importantly, these were not early-stage pathophysiological studies but rather late-stage clinical surrogates identified after decompensation had already occurred. These trials showed a critical lesson: digital monitoring without appropriate interpretation, clinical accountability, and therapeutic response does not change disease trajectory.

### 5.3. Implantable Hemodynamic Monitoring: Core Pathophysiology and Areas of Concern

The introduction of implantable pulmonary artery pressure monitoring, particularly via CardioMEMS technology, represented a key paradigm shift. High intracardiac filling pressures are evident days to weeks before symptomatic congestion, providing an optimal upstream target for intervention. Finally, the CHAMPION trial [50] established that pulmonary artery pressure–guided therapy effectively reduces HF hospitalizations in patients with NYHA class III HF. More recent studies using real-world post-approval registries and the Monitor-HF [51] program have confirmed these benefits in patients overall, including those with preserved ejection fraction, those aged 65 or older, and those with multiple comorbidities. Significantly, these benefits were not driven by increased device-related complications and were maintained over longer follow-up. CardioMEMS demonstrates the hallmark of effective digital medicine—monitoring works best when it taps into underlying pathophysiological signals and enables real-time treatment planning.

### 5.4. Structured Telemedical Care: TIM-HF, TIM-HF2

Outside of invasive hemodynamic monitoring, large-scale structured telemedicine initiatives have shown that noninvasive digital tools can also improve outcomes when integrated into broader care models. The TIM-HF [9,52] and TIM-HF2 [7,53] trials were significant in introducing telemonitoring in a central, physician-centered healthcare system. In TIM-HF2, patients with NYHA class II–III HF who underwent structured telemedical management experienced a meaningful reduction in all-cause mortality and unplanned cardiovascular hospital admissions [53]. Notably, the intervention was more than data collection. Daily observations were conducted alongside the continuous presence of healthcare providers, with an established plan for their response and a rapid therapeutic ramp-up if deterioration was detected. These results were corroborated by the Monitor-HF initiative, implemented in practice, which observed improvements in functional status, reductions in HF-related events, and continuity of care. Together, these studies show that digital medicine works when it redesigns how people receive care in the digital age, rather than when it simply digitizes what they already do.

### 5.5. TELESAT: The Therapeutic Power of the Human Link

Among contemporary studies, the French TELESAT [54] provides valuable insights into successful digital HF care. TELESAT showed a statistically significant reduction in all-cause mortality among participants in a national telemonitoring program. Still, its most important contribution comes from the discovery of the key importance of the patient–provider relationship. Subgroup analyses revealed that patients with limited digital literacy were, in general, more likely to be followed by frequent telephone calls rather than using smartphone apps or more sophisticated digital interfaces, and that a higher proportion were elderly. Strikingly, this subgroup showed mortality benefits similar to, or even greater than, those observed in cohorts in app-based monitoring. This discovery challenges the notion that better technology is an independent predictor of better outcomes. Instead, it indicates that digital medicine derives its therapeutic benefit, in significant part, from regular, structured communication between patients and healthcare providers. Simple technologies, if embedded in credible care relationships, can be as successful as high-tech digital systems.

### 5.6. Device-Based Monitoring and Arrhythmia Management

Implantable cardioverter-defibrillators (ICDs) and cardiac resynchronization therapy (CRT) devices continuously monitor rhythm disturbances, heart rate variability, physical activity, and surrogate markers of congestion. IN-TIME is one such trial that found that, by monitoring multiple metrics with a multiparameter device, centralized interpretation, and predefined clinical responses, all-cause mortality can be reduced [55]. In contrast, studies using standalone measures or not integrated into care pathways showed no benefit [56]. These conclusions reiterate that digital arrhythmia detection makes a clinical contribution only when situated within structured decision-making frameworks and systems that translate early signals into action sooner.

### 5.7. Practical Evidence: Implications and Health Systems Impact in Practice

Large-scale observational studies and national studies consistently demonstrate that implementing RPM programs reduces HF hospital admissions, emergency department visits, and length of hospital stay [57]. Crucially, there is real-world evidence of effectiveness in populations that are often poorly studied and rarely included in RCTs, such as the elderly, those with multiple diagnoses, and those with limited access to specialized HF centers. Health-economically, declines in acute care use offset the cost of digital infrastructure, helping ensure the sustainability of these models of care, provided an appropriate degree of reimbursement and a regulatory framework is in place [58].

### 5.8. Synthesis of Milestone Trials

Implantable hemodynamic monitoring in heart failure is the most direct pathophysiology-based digital medical intervention in the industry, focusing on intracardiac filling pressures that increase days to weeks prior to clinical decompensation. The CHAMPION trial proved that pulmonary artery pressure (PAP)–controlled intervention significantly reduced HF hospitalizations, demonstrating that upstream physiological surveillance can improve outcomes. Real-world data confirmed this reduction across diverse populations. In the GUIDE-HF trial, which included a broader HF group with prior hospitalizations or elevated natriuretic peptides, the primary endpoint was neutral overall, but analyses showed benefits before the pandemic, indicating external disruptions and implementation factors influence effects. The LAPTOP-HF trial, which targeted left atrial pressure, showed patient improvement but was halted early due to safety concerns, underscoring the importance of procedural safety and system readiness. The MONITOR-HF trial later confirmed the benefits of PAP-guided management in Europe, emphasizing that invasive hemodynamic monitoring is useful within integrated early intervention care models. Non-invasive remote patient management can serve as a scalable alternative. Early telemonitoring systems (e.g., TIM-HF, 2011) lacked mortality benefits, but TIM-HF2 improved outcomes by combining daily home assessments with a centralized clinical team, demonstrating that sustained engagement, not just technology, drives benefits. Large trials like Tele-HF and BEAT-HF showed no mortality or hospitalization benefit, largely due to poor data collection and response pathways. These results suggest that digital monitoring is effective when integrated into structured care with clear action plans, rather than merely for data transmission. Guidelines from ESC and ACC/AHA/HFSA recognize digital health as an enabling strategy essential for early detection, continuous care, and reduced hospitalizations, but emphasize that success depends on organizational readiness, trained personnel, proper data management, and clear escalation procedures. They advocate tailoring digital HF care to local healthcare systems and resources, emphasizing pragmatic implementation and value-based assessment.

### 5.9. Why Pathophysiology—Informed Digital Care Is the Game Changer

**Figure 8:**
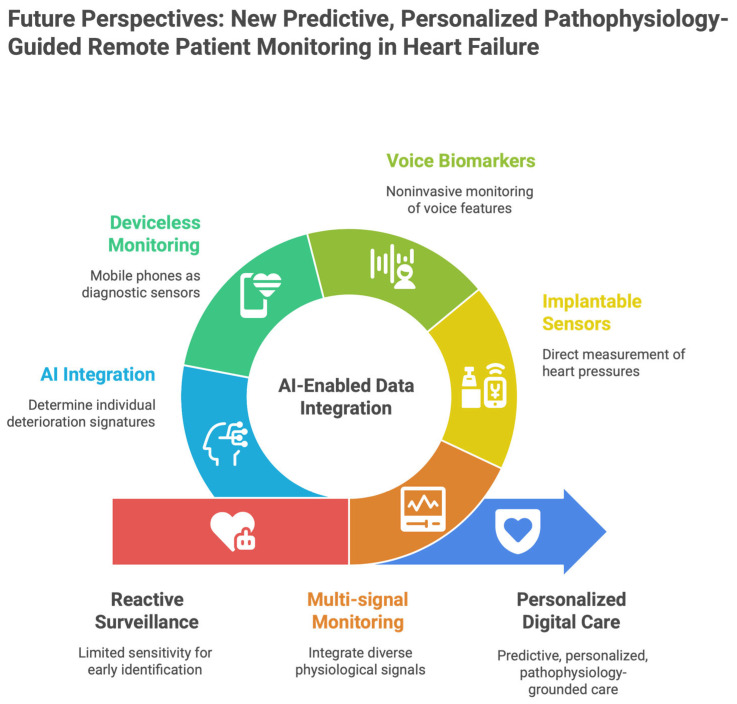
Future perspectives: New predictive, personalized pathophysiology-guided remote patient monitoring in heart failure.

The synthesis of evidence across trials and real-world studies consistently identifies a theme. Digital interventions fail when they are passive, disconnected, and fragmented from clinical decision-making [Figure 8]. They succeed when they are:

(a) Rooted in HF pathophysiology;
(b) Embedded in a multidisciplinary care pathway;
(c) Supported by well-trained healthcare teams;
(d) Based on ongoing patient-provider interaction;

Therefore, the efficacy of digital medicine in HF does not necessarily depend on the technology itself but rather on how effectively it optimizes care delivery to predict deterioration and respond promptly, thereby preserving the therapeutic alliance.

## 6. From Reactive to Predictive Care: The Future of Remote Monitoring in Heart Failure

Remote patient monitoring in heart failure (HF) has quickly evolved from a primarily reactive surveillance environment to a predictive, personalized, and continuously adaptive care-delivery paradigm. The next wave of digital medicine, built on data from a range of physiological signals, moves away from standalone devices toward entire ecosystems that capture multiple physiological signals, analyze them with artificial intelligence (AI), and translate insights into personalized medical practice [59].

### 6.1. Multi-Signal Monitoring to Multimodal Physiological Intelligence

**Figure 9:**
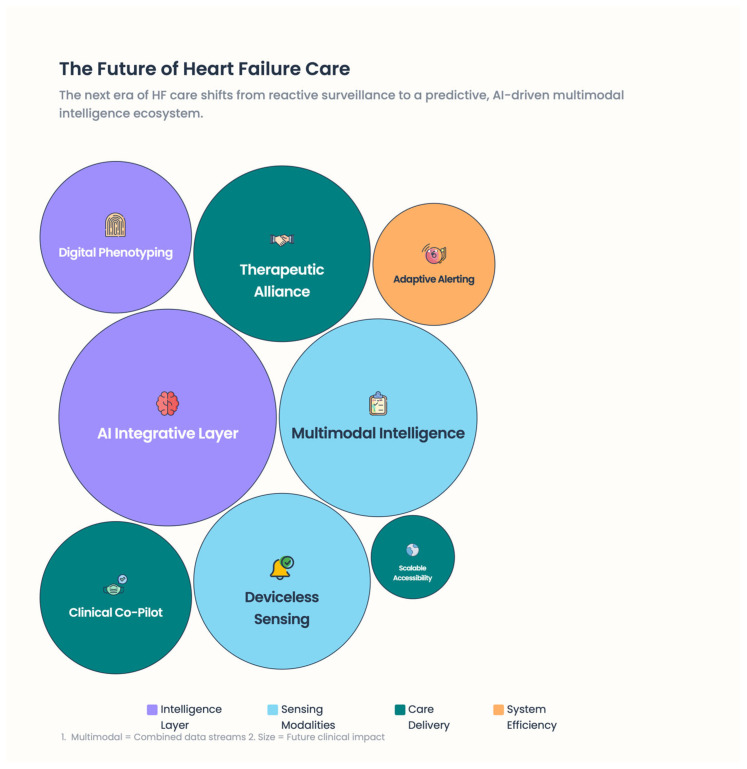
The future of heart failure care.

Early RPM systems primarily managed discrete parameters such as body weight, blood pressure, and heart rate. Although informative, these single measurements have limited sensitivity for early identification of HF degradation [60]. Next-generation RPM models emphasize multimodal data collection, integrating hemodynamic, autonomic, electrophysiological, behavioral, and contextual signals [61] [Figure 9]. Growing evidence indicates that combining multiple low-intensity signals may be more informative than high-fidelity measurements taken separately, because HF pathophysiology is complex and nonlinear. Although congestion, autonomic imbalance, arrhythmic changes, and neurohormonal activation often develop together, they remain asynchronous, making ongoing asynchronous follow-up essential rather than infrequent measures [62].

### 6.2. Implantable and Minimally Invasive Approaches: On Top of the Conventional Sensors

Although pulmonary artery pressure sensing is the most pathophysiologically direct implanted strategy, recently developed less invasive or minimally invasive techniques have broadened its application and reduced the complexity of the procedure. One of these technologies, the SeerlinQ™ system, targets left atrial pressure measurement and enables direct monitoring of left heart filling pressures [63]. Initial feasibility and first-in-human studies indicate that left atrial pressure monitoring is considered in addition to pulmonary artery pressure, or an alternative to in patients with preserved ejection fraction or atrial remodeling [64,65]. Long-term safety, signal stability, and clinical impact are being evaluated in continuing clinical trials. These trends are part of a broader shift toward pathophysiology-first monitoring that targets hemodynamic changes upstream rather than late symptomatic manifestations.

### 6.3. Voice-Based and Acoustic Digital Biomarkers

Voice-based technologies offer a promising, noninvasive approach to HF monitoring. Alterations in voice features, including pitch, amplitude variation, articulation, and respiratory modulation, can be associated with pulmonary congestion, fluid overload, and autonomic dysfunction. Various platforms, including those developed by Noah Labs and other researchers, have reported that voice-derived digital biomarkers can already predict HF decompensation days or weeks before presentation [66,67]. These systems are particularly appealing because they require no additional hardware and can support elderly or digitally limited patients. They can also be integrated into everyday telephone calls or virtual office visits. Voice analysis further supports a consistent theme across effective digital care: human interaction is the core activity, and technology enhances—not replaces—patient–provider interaction.

### 6.4. “Deviceless” Medical Monitoring Using Mobile Phones and Physiological Sensors

**Figure 10:**
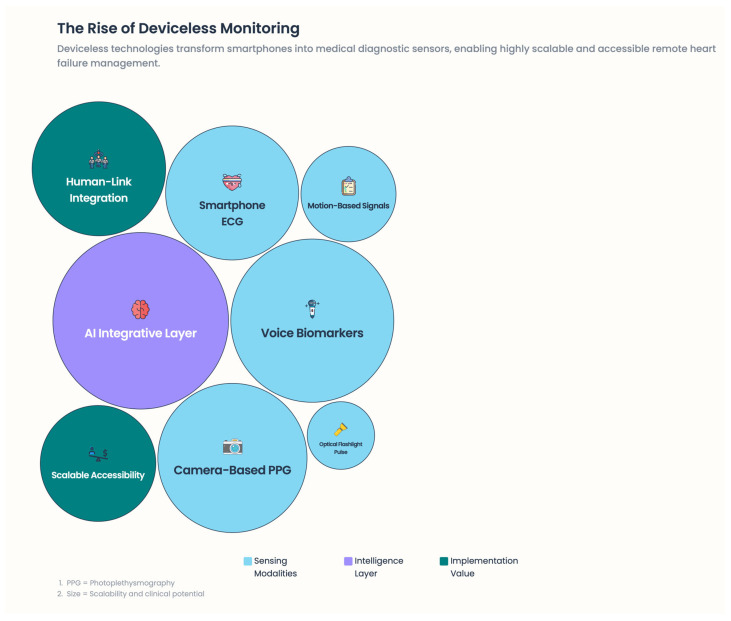
The rise of deviceless monitoring.

What has changed the paradigm in RPM is deviceless (device-light) technology, in which mobile phones serve as medical diagnostic sensors [Figure 10].

#### 6.4.1. Camera-Based Photoplethysmography (PPG)

By measuring subtle changes in skin color as a function of blood volume using a smartphone camera, camera-based PPG can estimate:

- Heart rate
- Heart rate variability
- Respiratory rate
- Peripheral perfusion indices

Platforms such as Binah.ai, FaceHeart, and IntelliProve have validated these methods against the reference standard and reported clinically acceptable accuracies, consistent with the reference [68,69,70]. Of further significance, contactless facial PPG measurements are available, making it usable and valuable, and its use is easy and adherent.

#### 6.4.2. Motion-Based Signals

Micro-movements associated with cardiac ejection and respiratory mechanics, as measured by accelerometers and gyroscopes, can also be recorded on a smartphone. Companies producing smartphone-assisted ballistocardiography have shown that such devices can be used to time cardiac intervals and assess cardiac output trajectories [71,72,73].

#### 6.4.3. Measurements Using Optical and Flashlight Devices

Several applications can obtain pulse waveforms and pulse transit time surrogates, such as smartphone flashlights and cameras. Using these methods, indirect estimates of vascular tone and autonomic balance can be made [74]. Together, these methods reduce barriers to adoption, particularly for low-resource or unreceptive population groups that are reluctant to wear wearables.

### 6.5. Smartphone-Embedded ECG and Arrhythmia Surveillance

Smartphone-enabled ECG monitoring technologies, from single-lead to multi-lead, are increasingly used to detect arrhythmias in patients with HF. These devices are suitable for detecting atrial fibrillation, conduction abnormalities, and ventricular ectopy in the early stages and enable prompt therapeutic procedures. Smartphone ECGs, when integrated with symptom reporting and physiologic context, enable arrhythmia-aware HF treatment and closely reflect the Special Issue’s emphasis on arrhythmias [75].

### 6.6. AI as the Integrative Layer

The real transformative potential of future RPM lies not in individual technologies but in AI-enabled data integration. Machine learning models can use heterogeneous data streams (hemodynamics, voice, motion, ECG, behavior) to determine individual deterioration signatures. Implement adaptive alert thresholds—decision-making for titration of therapy. AI does not substitute for doctors but acts as a clinical co-pilot, filtering noise, prioritizing risk, and enabling early, accurate treatment based on a patient’s path [76].

### 6.7. Personalized, Relationship-Driven Digital Care

**Figure 11:**
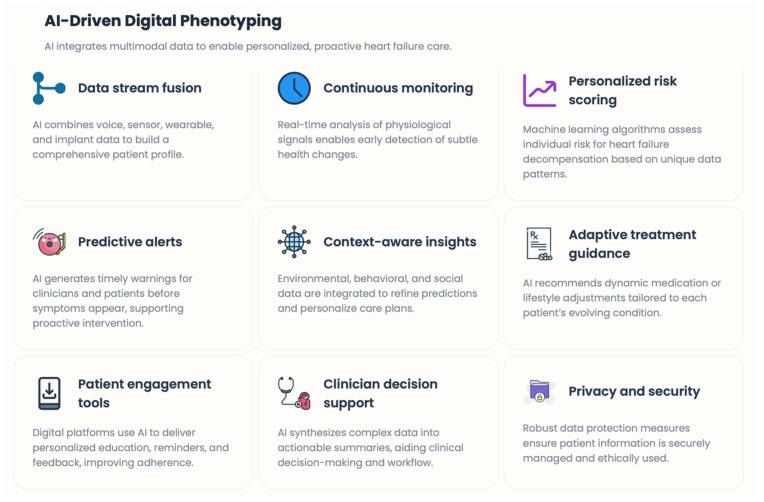
AI-driven digital phenotyping.

Future RPM models will increasingly rely on patient-specific digitized phenotyping and move away from population-based algorithms, as HF progression is known to be individualized [77] [Figure 11]. Just as importantly, evidence shows that technology alone is not enough. These digital care models retain and enrich the therapeutic alliance between patients and health care providers. Multimodal sensing, smartphone technology, AI analytics, and structured human oversight together describe a new era in HF management—one that is predictive, personalized, and deeply grounded in pathophysiology.

### 6.8. Digital Monitoring Technologies: Clinical Utility, Limitation and Failure Modes in Heart Failure

Structured, non-invasive remote patient management, such as TIM-HF2, shows positive outcomes when daily data are integrated into a care model with clear escalation pathways. Evidence suggests fewer hospital visits and potential mortality benefits, underscoring the value of low-cost surveillance and proactive management. However, these programs rely heavily on organizational infrastructure, telemedicine teams, and physician oversight. Without these, monitoring becomes passive, affecting adherence and scalability, especially if patients feel overwatched or if feedback is weak.

Implantable pulmonary artery pressure sensors offer direct info on congestion, enabling preemptive therapy adjustments. Studies show reductions in hospitalizations and improved quality of life, especially for recurrent decompensation. Limitations include invasiveness, expense, and a need for specialized skills and infrastructure. Benefits decrease if data are reviewed infrequently or if clinicians lack authority for therapy changes. Access and reimbursement issues also limit broad use.

ICD/CRT devices provide ongoing data on rhythm and other parameters, which, when integrated into structured workflows, can detect arrhythmias and early decompensation, improving outcomes. But single parameters may give false alarms, and alerts depend on human review. These systems are only for patients with implantable devices and do not scale well for larger populations.

Wearables and smartphone tracking can monitor heart rate, rhythm, activity, and sleep, enabling early decline detection, especially with symptom reporting. Challenges include variable data quality, reliance on surrogate endpoints, and the potential for noise over actionable data. Costs, digital literacy, and engagement also create inequities.

Deviceless monitoring (Camera-PPG, voice analysis, ballistocardiography) offers accessible options for older or less tech-savvy patients. Early studies show potential for detecting congestion and fatigue, but evidence is limited, and environmental factors can affect accuracy. Without validation and oversight, these may overpromise benefits while leaving unresolved regulatory and privacy issues.

Success across modalities depends on actionable signals, workflow integration, and human oversight. Innovation alone does not ensure scalability or equity—cost, simplicity, and training are key. Digital programs are ineffective if monitoring is passive, thresholds are unclear, responsibility is lacking, or staffing falls short.

### 6.9. Parameter Choice and Dimensionality Concerns

Although remote patient monitoring enables the collection of multiple physiological and behavioral parameters, increasing the number of monitored parameters does not necessarily improve clinical decision-making. On the other hand, too much dimensionality can induce redundancy, noise, reduced interpretability, and an operational burden—termed by experts the “curse of dimensionality”—especially when the models are insufficiently calibrated or lack clinical validation. Adequate RPM systems focus on a small but physiologically relevant and clinically meaningful set of parameters rather than indiscriminate data accumulation. In this regard, multiparametric monitoring must be designed around feature selection and prioritization and must consider individual thresholds anchored to patient-specific baselines, with transparency in clinical interpretability rather than opaque “black-box” automation. For instance, advanced analytics and artificial intelligence have the potential to assist in this process by incorporating heterogeneous signals into simplified risk trajectories which are made accessible and the application of concepts such as “management by exclusion,” whereby individuals who maintain physiological characteristics defined by an individual need minimal interventions from the clinician, and the clinical focus becomes on individuals presenting out-of-range or developing deterioration forms. This balance of informative richness and practical usability aligns digital monitoring with real-life clinical workflows.

## 7. Clinical Implications and Implementation in Heart Failure Care Pathways

Constraints and limitations in the evidence available to date. The evidence base suggests that digital monitoring for heart failure has no universally beneficial effect. Instead, clinical value emerges when digital tools are integrated into systematic care pathways that capture physiologic information and convert it into early treatment. Trial-proven advantages are already available for some techniques, including structured remote patient care programs and invasive hemodynamic monitoring, while other methods should be considered adjunctive or investigational. Future innovation, then, should focus less on the proliferation of monitoring technologies and more on the science of implementation: setting actionable limits, streamlining care workflows, and ensuring that digital monitoring is consistent with value-oriented health-system goals. Until additional confirmation is obtained, developing strategies for these new formats will require cautious prospective assessment and avoiding overgeneralization beyond the evidence base.

It also means that the adoption of digital medicine and remote patient monitoring (RPM) in heart failure (HF) is more than a technological innovation and more than a question of integration into established clinical pathways and of redefining care delivery. The results from clinical trials and real-world programs repeatedly suggest that the benefits of digital tools are significant when incorporated into a structured, multidisciplinary clinical approach to HF that converts physiological data into timely clinical action [78].

### 7.1. An Episodic to Continuous Perspective of Heart Failure Care

**Figure 12:**
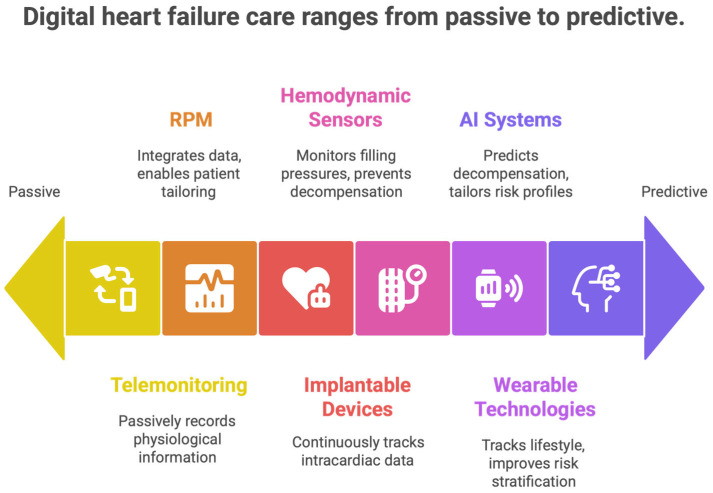
Digital heart failure care ranges from passive to predictive.

Conventional HF management is predominantly episodic and reactive, relying on outpatient care and hospital admission for worsening signs. Digital medicine enables clinicians to move toward continuous, longitudinal care by monitoring real-time physiological trends, symptom evolution, and behavioral patterns. This transition allows for early detection of decompensation, proactive optimization of guideline-directed medical therapy (GDMT), and prevention of acute events [Figure 12]. Clinically, this is a paradigm shift: HF care now focuses on restoring trajectory rather than crisis management [79]. Real-time assessment of congestion markers, heart rate dynamics, rhythm disturbances, and patient-reported symptoms enables timely interventions during the early preclinical stage of HF progression, when therapeutic responses are most effective [80].

### 7.2. Reconfiguration of the Role of the Multidisciplinary Heart Failure Team

**Figure 13:**
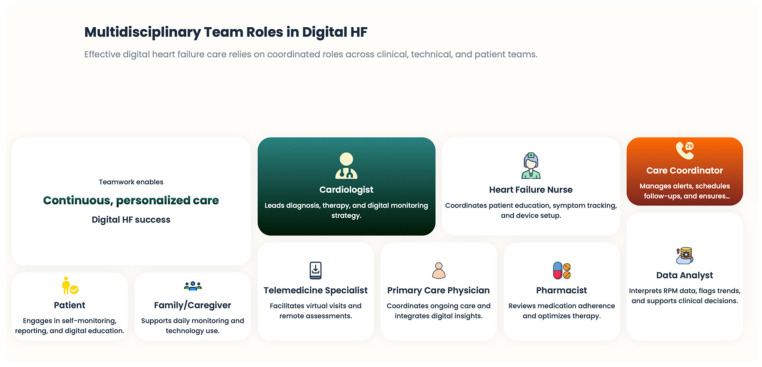
Multidisciplinary team roles in digital HF.

RPM integration requires an evolution in HF multi-disciplinary teams. Medical practitioners, HF nurses, colleagues in health care, and digital care coordinators work together to interpret data, speak with patients, and make changes to care delivery [Figure 13]. Teams of telemedicine professionals have proven remarkably successful in managing alerts, triaging risks, and promptly escalating care. Crucially, it is here that the digital health environment plays a complementary role, one that does not detract from clinicians’ expertise but enhances it, in providing individualized care [81]. Structured workflows, set response algorithms, and protocols for escalation are necessary to mitigate alert fatigue and to translate data into clinically relevant action, particularly digital insights into meaningful behavior that meet or exceed expectations, in accordance with our need for these findings.

### 7.3. The Integrated Model Between Levels of Clinical Care

Primary care, specialist HF clinics, and hospital-based care, as well as bridging gaps between the health and healthcare systems, are among the greatest clinical applications of RPM. Digital tools can support seamless information transmission between these levels and promote continuity, for example, during transitions in care (hospital discharge, medication titration, disease progression). The high-risk period for HF patients is the post-discharge period. RPM programs that start early or immediately after discharge have been shown to reduce early rehospitalizations by enabling close monitoring, early detection of congestion, and strengthening medication adherence and self-care behaviors.

### 7.4. Patient Engagement and Self-Management as Integral Components of Healthcare

Active patient participation is needed for digital medicine integration to be effective. By offering feedback, education, and confidence in their self-management, RPM platforms can enable patients to be more involved in their own care. Yet the level of technological complexity should be adapted to patients’ abilities, preferences, and health literacy. Data from clinical trials also show that effective patient–provider communication via a smartphone (or some combination of devices) can be a key success factor. Hence, pathways for digital care must emphasize accessibility, inclusivity, and personalization, as digital technology development must not be the same for all [82].

### 7.5. Contribution to Arrhythmia Surveillance in Heart Failure Pathways

Arrhythmias are among the leading causes of HF morbidity and mortality. Rhythm monitoring can also facilitate the early identification of atrial fibrillation, ventricular arrhythmias, and conduction abnormalities, which can trigger decompensation or precipitate sudden cardiac events [21]. When added to RPM, rhythm monitoring enables the timely initiation of anticoagulation, device reprogramming, or pharmacological interventions when continuous or intermittent rhythm assessment is linked to actionable care pathways. This approach allows arrhythmia management to be integrated with the broader HF disease management strategy, meaning arrhythmias are not treated in isolation; instead, they can develop over time and be managed alongside HF disease control.

### 7.6. Health System and Policy Implications

At the health system level, implementing digital HF care pathways affects financial and funding decisions, reimbursement strategies, and resource planning. Evidence of declines in hospitalizations and acute care utilization aligns with the economic justification for reimbursed RPMs, particularly in systems with elevated HF prevalence and constrained inpatient capacity. Eligibility criteria, data governance, and quality metrics will be necessary to make access more equitable and care more consistent across regions and populations [83].

### 7.7. Key Clinical Takeaways

When efficiently connected to HF care pathways, digital medicine and RPM, this would:

- Enable earlier intervention throughout the trajectory of HF disease.
- Proactively optimize GDMT.
- Enhance collaboration among care environments.
- Increase patient care and self-management.
- Minimize hospitalizations and healthcare utilization.

The clinical utility of digital HF management is not in the technology but in its ability to enable patient-centered, pathophysiology-informed, and constantly adapting models of care.

### 7.8. Challenges in Monitoring and Operation

The frequency and intensity of telemonitoring in heart failure may vary widely and depend on the technology used and the mode of care. In organized nonintrusive remote patient management protocols, monitoring involves daily transmission of body weight, blood pressure, heart rate, and standardized symptom scores, although lower-risk participants might be monitored several times per week. Implantable cardiac electronic devices (ICD/CRT) can continuously monitor rhythm, activity, and selected physiological parameters with scheduled remote interrogations and event-triggered alerts. Implantable hemodynamic sensors typically involve daily or near-daily pressure uploads to facilitate anticipation of necessary therapeutic manipulation. Wearable and deviceless tools (e.g., smartphone-based photoplethysmography or voice analysis) can work via passive continuous sampling or patient-initiated periodic measurements. There are distinct “hidden challenges” that affect both effectiveness and scalability across all models. These include alert fatigue and signal noise that may mask actionable deterioration, increased workload in the absence of dedicated monitoring teams, variable long-term patient adherence, and barriers to digital literacy—especially in elderly populations. Additional challenges include data overload, cybersecurity and governance demands, incorporation into electronic medical records and established care pathways, and ambiguity about reimbursement and clinical responsibility (i.e., who reviews data, when, and how escalation happens). These considerations highlight that telemonitoring is not simply a technological intervention but a resource-dependent clinical service that must be delivered within a structured operational framework.

## 8. Restrictions, Moral Consideration, and Barriers to Implementation

**Figure 14:**
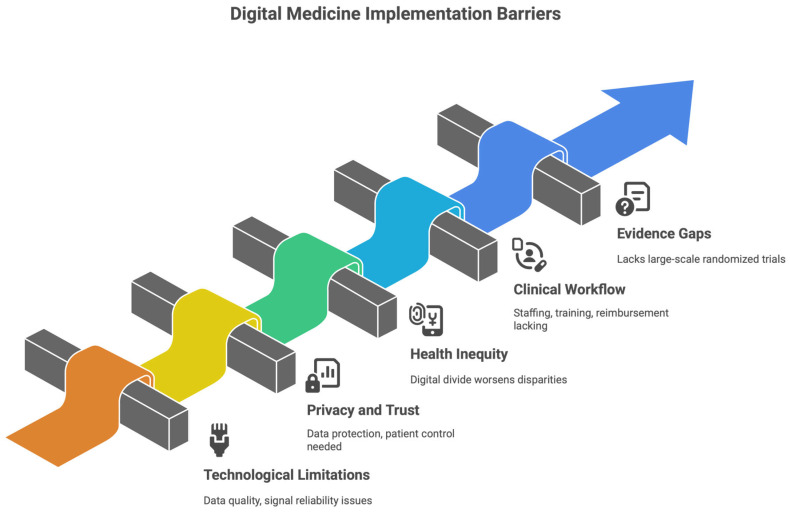
Digital medicine implementation barriers.

Notwithstanding the mounting evidence of the advantages of digital medicine and remote patient monitoring (RPM) in the clinical management of heart failure (HF), some caveats and challenges need to be addressed if responsible, effective, and equitable practice is to be realized. These barriers [Figure 14], however, are not only technical; they go beyond technology to include ethical, organizational, regulatory, and human obstacles, providing evidence that the digital transformation of HF care is part and parcel of health care, as much a systems challenge as a scientific problem.

### 8.1. Limitations Caused by Technological and Information-Oriented

The continuous acquisition, transmission, and interpretation of digital HF data are critical to management. Data quality, signal reliability, and interoperability between devices and electronic health record systems are significant shortcomings. Inconsistencies arising from hardware, software, and algorithmic design can add complexity and challenge clinical interpretation and decision-making. The increased demand for patient-generated health information also risks information overload and alert fatigue, especially in regions where thresholds are ill-specified or algorithms are under-validated. Without intelligent data filtering and ranking, clinicians may receive excessive non-actionable alerts, which can harm clinical effectiveness and safety [84].

### 8.2. Issues Around Privacy, Autonomy, and Trust

Ethics in digital technology for HF care, including ethical issues related to data protection, safety, and patient control, needs attention. Continuous monitoring generates sensitive health data that must be kept secure to prevent unauthorized access, breaches, and misuse. Compliance with data protection laws and the protection of patient trust must be part of the baseline for maintaining a sustainable digital care model. Patient autonomy is as vital as patient autonomy. Although RPM’s sole goal is to improve care, aggressive surveillance or the implementation of monitoring strategies not communicated effectively may result in anxiety, perceived loss of control, or disengagement. Open communication about the purpose, scope, and limitations of monitoring is thus vital [85].

### 8.3. Health Inequity—The Digital Divide

The most significant risk of increasing health inequities in digital HF care is that they could worsen. Complex digital platforms can also disadvantage older adults and patients with low digital literacy, lower socioeconomic status, or limited access to technology [86]. These disparities may be mitigated through simplified interfaces, human-mediated communication (e.g., telephone-based follow-up), and appropriate adaptations, which, once again, must be designed for in RPM programs, as evidenced by real-world evidence. Failure to bridge the digital divide will lead to selective benefits for technologically proficient populations, excluding those at the highest risk of adverse HF outcomes.

### 8.4. Clinical Workflow and Staff Challenges

Adopting RPM is central to repositioning clinical workflows for improvement. Intensive teams, clear lines of responsibility, and clear escalation pathways are necessary to translate the digital signal into a prompt for therapeutic intervention. Without sufficient staffing, training, and reimbursement, digital HF interventions can become either unprofitable or underappreciated. In addition, clinicians should develop new skills in digital literacy, data interpretation, and remote communication. To develop these competencies, digital devices like those used by digital health professionals are expected to be used remotely. This evolution is a cultural change in HF care—one that will necessitate institutional support and ongoing professional development [87].

### 8.5. Sustainable Development Goals Alignment

Digital medicine and remote patient monitoring in heart failure directly align with several United Nations Sustainable Development Goals (SDGs). More directly, they contribute to achieving SDG 3 (Good Health and Well-Being) by facilitating earlier detection of deterioration, preventing avoidable hospital admissions, and supporting the continuity of care for patients with chronic non-communicable diseases. By providing remote access to specialist-level expertise, digital HF care further supports the goal of SDG 10 (Reduced Inequalities), particularly in rural or underserved regions, provided the delivery is inclusive and not solely dependent on high-cost technology and advanced digital literacy. Furthermore, scalable remote monitoring systems and interoperable digital platforms are being developed, contributing to SDG 9 (Industry, Innovation and Infrastructure) in support of robust health infrastructure and health-technology ecosystems. Lastly, the effective implementation of digital HF care depends on cooperation among health care systems, technology providers, academia, and policymakers, in line with one of the goals of SDG 17 (Partnerships for the Goals). Importantly, digital medicine can both reduce and exacerbate health disparities, so aligning with the SDGs hinges on policy frameworks that prioritize equity, accessibility, and workforce capacity, alongside innovative technology.

### 8.6. Evidence Gaps and Regulatory Considerations

Even as the literature on digital HF interventions continues to grow, key obstacles remain. Many technologies lack large-scale randomized trials demonstrating long-term effects on mortality, arrhythmia burden, or disease progression. Technological advances often move quickly, outpacing regulatory oversight, creating uncertainty about validity, liability, and reimbursement. As regulatory frameworks evolve to reflect innovation while maintaining patient safety, the challenge will be to promote an evidence-based approach to clinical adoption while minimizing the risk of early, unproven use.

### 8.7. Accessibility and Cost Issues

Digital medicine in heart failure has very different economic footprints, depending on the mode of monitoring employed and the level of technological complexity. On the low-cost side, remote care could be provided through structured telephone follow-up, accompanied by easily accessible non-invasive measures such as body weight, blood pressure, heart rate, and symptom scores, which often rely on patients’ existing devices. Examples of intermediate-cost models include nurses running remote patient programs with support from mobile applications, dashboards, or centralized monitoring infrastructure. More expensive possibilities include implantable hemodynamic sensors and comprehensive digital platforms that might improve physiological fidelity but demand additional upfront investment and specialized infrastructure. There are multiple challenges in achieving equity of access to digital heart failure care, especially for people of low socioeconomic status. These include the cost of smartphones or wearables, poor internet access in rural areas, differences in reimbursement and coverage policies, digital literacy—especially among older adults—language and access design barriers, workforce capability to monitor and respond to incoming data, and a lack of national governance mechanisms and interoperability standards. The only available and pragmatic solution is a stepped-care model, where many patients use low-cost, scalable monitoring, and more intensive, resource-intensive technologies are used for higher-risk populations. Such a stratified deployment links investment in digital health to clinical risk, enhances the system’s sustainability, and improves population-wide access.

## 9. Conclusions

Digital medicine and RPM are transforming heart failure (HF) management from reactive to proactive personalized care. Evidence from trials, real-world studies, and telemedicine programs shows that digital tools can reduce hospitalizations and costs and improve survival and quality of life, especially when integrated into a structured care continuum. Success depends not just on technology but also on capturing accurate physiological signals, receiving feedback from the healthcare team, providing timely interventions, and maintaining patient–provider contact, which is crucial for older, less tech-savvy patients. Emerging tools like AI, voice monitoring, smartphone sensing, and digital biomarkers expand RPM, offering personalized, scalable, and accessible HF care. Their clinical value hinges on validation, integration, and ethical oversight. The future combines advanced digital sensing with human judgment, multidisciplinary care, and patient empowerment, promising a significant leap forward in HF treatment.

### Limitations

This narrative review has several significant limitations that deserve explicit attention. First, the evidence base in the field of digital health and remote patient monitoring in heart failure is heterogeneous with respect to study design, patient demographics, monitoring intensity, and reported endpoints, making direct comparisons across studies challenging. Second, monitoring of protocol adherence and digital literacy among patients, especially among the elderly, greatly varies, thereby influencing efficacy and generalizability. Third, clinical benefit is highly implementation-dependent, requiring sufficient staffing, established workflows, and escalation capacity, none of which are universally applicable across health systems. Fourth, while emerging deviceless biomarkers (e.g., voice, camera-based PPG, ballistocardiography) are promising, robust hard-outcome data remain limited, and most evidence is derived from feasibility or observational studies. Lastly, pragmatic randomized trials and health-economic evaluations across diverse health care settings, particularly in middle-income and resource-constrained areas, are required to help define cost-effectiveness and value in the real world.

## Figures and Tables

**Table 1:** Summary of key randomized trials and real-world studies of digital heart failure monitoring, stratified by technology category and clinical endpoints.

Technology Category	Key Studies/Programs	Monitoring Parameters	Care Model and Response	Main Clinical Endpoints	Key Findings/Interpretation
Structured non-invasive remote patient management	TIM-HF (2011); TIM-HF2 (2018); extended follow-up TIM-HF2 (2020)	Daily body weight, blood pressure, heart rate, symptom/status questionnaires	Central telemedical center with trained healthcare professionals; predefined escalation; physician oversight; proactive patient contact	Composite endpoint (days lost due to unplanned CV hospitalization or death), all-cause mortality	TIM-HF showed neutral mortality effect; TIM-HF2 demonstrated a significant reduction in primary composite endpoint and a signal toward lower all-cause mortality. Benefit driven by structured workflow and continuous patient–HCP interaction rather than technology alone
Non-invasive telemonitoring (less structured)	Tele-HF; BEAT-HF	Weight, symptoms, basic vitals	Passive or semi-passive data transmission; variable response intensity	Mortality, HF hospitalizations	Largely neutral results; highlighted limitations of monitoring without clear action pathways and sufficient response intensity
Implantable hemodynamic monitoring (PAP sensors)	CHAMPION; GUIDE-HF; MONITOR-HF	Pulmonary artery pressure	Pressure-guided therapy adjustments within structured HF care programs	HF hospitalizations, quality of life, composite endpoints	Consistent reduction in HF hospitalizations; MONITOR-HF showed significant improvement in health-related quality of life. Reinforces value of upstream physiological congestion detection when embedded in care pathways
Implantable electronic cardiac device diagnostics (ICD/CRT)	IN-TIME; multiple real-world registries	Heart rhythm, AF burden, ventricular arrhythmias, thoracic impedance, activity, HRV	Remote device interrogation with centralized review and escalation	Mortality, HF worsening, arrhythmia detection	Multiparametric device diagnostics improve outcomes when reviewed systematically and linked to timely intervention; isolated parameters alone are insufficient
Wearables and smartphone-based monitoring	Observational studies; pilot trials	PPG-derived HR/rhythm, activity, sleep patterns	Variable; often patient-initiated or app-based	Surrogate endpoints, early deterioration detection	Promising for scalable monitoring; evidence supports signal detection but requires integration into clinical workflows for outcome impact.
Deviceless monitoring (camera, voice, BCG)	Early feasibility and real-world pilots (e.g., voice analysis platforms, camera-PPG, BCG)	Facial PPG, voice biomarkers, respiratory patterns, mechanical cardiac signals	Low-burden patient interaction; often combined with phone/video follow-up	Early detection of congestion or deterioration (exploratory)	Emerging technologies show potential for elderly and low-resource settings; require validation and multimodal integration to improve robustness
Connected-care real-world programs	TELESAT (France); regional RPM programs	Mixed non-invasive parameters; phone-based follow-up	Strong emphasis on human interaction; phone contact often primary interface	All-cause mortality, HF hospitalizations	Mortality benefit observed even in digitally less literate patients contacted primarily by telephone, underscoring the central role of human connection.

## Data Availability

No new data were created or analyzed in this study. Data sharing is not applicable to this article.
